# Spotlight on the Life Cycle of Acrylamide-Based Polymers Supporting Reductions in Environmental Footprint: Review and Recent Advances

**DOI:** 10.3390/molecules27010042

**Published:** 2021-12-22

**Authors:** Olivier Braun, Clément Coquery, Johann Kieffer, Frédéric Blondel, Cédrick Favero, Céline Besset, Julien Mesnager, François Voelker, Charlène Delorme, Dimitri Matioszek

**Affiliations:** SNF SA, ZAC de Milieux, 42160 Andrézieux-Bouthéon, France; obraun@snf.com (O.B.); ccoquery@snf.com (C.C.); Johann.kieffer@snf.com (J.K.); fblondel@snf.com (F.B.); cfavero@snf.com (C.F.); cbesset@snf.com (C.B.); jmesnager@snf.com (J.M.); fvoelker@snf.com (F.V.); cdelorme@snf.com (C.D.)

**Keywords:** water soluble polymer, sustainable process, macromolecular engineering, raw material sourcing, reduced industrial footprint, polymer fate assessment, polyacrylamide handprint

## Abstract

Humankind is facing a climate and energy crisis which demands global and prompt actions to minimize the negative impacts on the environment and on the lives of millions of people. Among all the disciplines which have an important role to play, chemistry has a chance to rethink the way molecules are made and find innovations to decrease the overall anthropic footprint on the environment. In this paper, we will provide a review of the existing knowledge but also recent advances on the manufacturing and end uses of acrylamide-based polymers following the “green chemistry” concept and 100 years after the revolutionary publication of Staudinger on macromolecules. After a review of raw material sourcing options (fossil derivatives vs. biobased), we will discuss the improvements in monomer manufacturing followed by a second part dealing with polymer manufacturing processes and the paths followed to reduce energy consumption and CO_2_ emissions. In the following section, we will see how the polyacrylamides help reduce the environmental footprint of end users in various fields such as agriculture or wastewater treatment and discuss in more detail the fate of these molecules in the environment by looking at the existing literature, the regulations in place and the procedures used to assess the overall biodegradability. In the last section, we will review macromolecular engineering principles which could help enhance the degradability of said polymers when they reach the end of their life cycle.

## 1. Introduction

Humankind is facing a global crisis that is unprecedented in terms of scale, reach and magnitude, with possible dramatic changes to the way many people live today. The equation to solve is complex and multiparametric: how can we address the urgent climate change issue, whose unfolding has been triggered by the massive use of fossil fuels, and transition towards cleaner, reliable, and cheap energy sources in a world with constrained resources, in a limited time?

To tackle this issue, science has one very important role to play, and all disciplines should collaborate to provide fresh and innovative ideas to perpetuate mankind’s sustainable journey on Earth. As perfectly stated by Matlin [1], “chemistry is one of these ‘platform’ or ‘central’ sciences, underpinning fundamental aspects of a range of established and emerging sciences including biochemistry, nanoscience, molecular and synthetic biology, physics and soft condensed-matter physics; as well as many major practical advances seen in such fields as agriculture, biotechnology, energy, ecology, the environment, genetics, information technology, materials and medicine; and the dramatic rises in overall human health and well-being during the past two centuries”. It is also one of these sciences whose importance and impact are hidden for many people in their daily lives, even though it is everywhere: from the water we drink to the crops we grow. 

Among the large number of molecules provided by science, acrylamide-based polymers actively participate in improving daily life as markets are closely related to the growth of the population and the scarcity of resources: water, ore, food mineral, pulp and hydrocarbons [2,3,4]. The global polyacrylamide market size was valued at USD 4.5 billion in 2018 and is projected to expand at a CAGR of 6.2% from 2019 to 2025 [5]. In volume, this represents an output of about 2.5 million tons.

Polyacrylamide, or PAM, is made from the acrylamide monomer. Although PAMs are not toxic, the starting building block, acrylamide, is one of the substances of very high concern (SVHC) and is included in the candidate list for authorization [6,7,8]. This implies that the industry must comply with strict regulations to control the residual level of monomers in finished products [9]. This is especially the case when PAMs are used as a flocculant in drinking water, where the maximum accepted level is ranging from 1 to 5 ppb [9]. Fortunately, acrylamide is easily hydrolyzed and biodegraded when disposed in an aquatic environment due to its high water solubility and its ability to be degraded by micro-organisms [10,11,12].

This paper will focus on the life cycle of acrylamide-based polymers and intends to provide improvement ideas for the use of these water-soluble polymers throughout the value chain by considering five pillars, inspired by the “green chemistry” concept: (1) raw material sourcing, (2) reduced industrial footprint, (3) helping end user customers to reduce their energy/water consumption, (4) regulatory compliance and (5) understanding the end of life, the fate and the behavior of the polymers when released into the environment. The “green chemistry” concept emerged in the early 1990s with foundation of the Green Chemistry Institute, co-founded by Dr Joe Breen and Dr Dennis Hjeresen. Few years later, the 12 Principles of Green Chemistry by Anastas and Warner [13] was groundbreaking, outlining a framework for making a greener chemical industry and continuing nowadays to guide academic and industrial scientists. Among these principles, seven are readily applied to the manufacture of PAM:

1. Prevention: Prevent waste formation instead of treating or cleaning waste after it has been created. Roger Sheldon described the concept of the E-factor as a dimensionless number which measures the weight of waste coproduced with the weight of desired product [14].
2. Atom economy: This concept was developed by Barry Trost in a way to shift initial paradigms focusing on measuring reaction efficiency by calculating the yield to the efficiency of the incorporation of atoms from reactants to desired product [15].
3. Less hazardous chemical syntheses: Synthetic methods should be designed to use and generate substances that possess little to no toxicity to human health and environment.
4. Catalysis: Catalytic reagents (as selective as possible) are superior to stoichiometric reagents. It lies in the prevention, atom economy and design for energy efficiency principles previously described where the catalyst can increase the selectivity and kinetic energy of a reaction while minimizing the waste generation and reaction temperature. The most exquisite catalysts are the enzymes which are particularly effective at enhancing selectivity with simple or complex substrates under mild conditions.
5. Safer solvents and auxiliaries: The use of auxiliary substances (e.g., solvents, separation agents, etc.) should be made unnecessary wherever possible and innocuous when used.
6. Design for energy efficiency: Energy requirements should be recognized for their environmental and economic impacts and should be minimized. Synthetic methods should be conducted at ambient temperature and pressure.
7. Use of renewable feedstocks: A raw material or feedstock should be renewable rather than depleting whenever technically and economically practicable.

After discussing the current and new raw material sourcing possibilities, we will detail improvements in the manufacturing processes and continue with the regulatory aspects surrounding the use of polyacrylamides. We will finish with the end uses of these polymers and how they can help reduce the environmental footprint of anthropic activities.

## 2. A Review of Raw Material Sourcing Options

### 2.1. Raw Materials

Key raw materials for PAM and derivatives are monomers such as acrylamide, acrylic acid and [2-(acryloyloxy) ethyl] trimethylammonium chloride. Today, these molecules are sourced from fossil raw materials and, more especially, are derivatized from the C3-value chain with the transformation of propylene.

- Acrylamide

Acrylamide results from the hydration reaction of acrylonitrile. An old chemical pathway involved the hydration of the nitrile group with sulfuric acid via the acrylamide sulfate intermediate [16], the latter being further neutralized to release free acrylamide while coproducing sodium sulfate salt as byproduct. Back in the 1970s, copper catalyst was introduced in this reaction to avoid obtaining the salt byproduct [17]. However, this reaction still had several drawbacks such as the temperature of the reaction, the uncomplete acrylonitrile conversion (forcing the evaporation and recycling of unreacted acrylonitrile), the need to remove the copper catalyst (generating waste) and generation of undesired byproducts affecting the overall quality and performance of acrylamide.

More recently, a bioprocess has been developed which uses an enzyme to convert the nitrile function to an amide one. Multiple genetic strategies enabled the evolution of a recombinant *Rhodococcus rhodochrous* nitrile hydratase (NHase) to enhance its activity, stability and resistance to the reaction mixture [18,19,20]. With this process, the conversion of acrylonitrile to acrylamide reaches 100% with 100% selectivity and no waste generation. The so-called copper catalyst process is now almost phased out and the bioprocess is the main one currently used by the industry. The industrial application of NHase in hydrating acrylonitrile to acrylamide is one of the most successful biocatalyst cases, leading to a worldwide production of more than 1 million tons of acrylamide per annum (on a dry basis). The successful use of this enzymatic technology is a good example of how the footprint (atom economy, waste, less hazardous catalysis, energy efficiency, etc.) of a chemical synthesis can be reduced while enabling, at the same time, the production of large volumes at an economical level.

- Acrylic acid and derivatives

Acrylic acid is a key monomer with an estimated annual production capacity of about 6 million tons. The major end uses of acrylic acid and derivatives are superabsorbent, polymeric flocculants, dispersants, paints, coatings and adhesives. Acrylic acid is mostly produced from propylene, which involves heterogeneous catalytic oxidation of the latter in the vapor phase with air and steam at temperatures ranging from 260 to 370 °C. A two-step process via acrolein is preferred, achieving about 90% overall yield [21,22,23]. The acrylic acid produced during the reaction is purified by rectification or melt–recrystallization. Heavy ends are generated and considered as waste to be disposed of or incinerated along with wastewater.

[2-(Acryloyloxy) ethyl] trimethylammonium chloride is a well-known cationic ester derivative used to make polymers applied in many industries such as water treatment, papermaking, home and personal care and others. This monomer is mostly produced by a catalyzed transesterification reaction between alkyl acrylate and dimethylaminoethanol, in a solvent with either organo-tin [24,25] or tetra alkyl titanate [26] compounds to limit the formation of impurities such as Michael adducts formed with low molecular weight alcohols (such as methanol). At the end of the reaction, the catalyst is separated from the reaction mixture and recycled if its activity is high enough to limit the formation of impurities. 2-(Dimethylamino) ethyl acrylate is further quaternized with alkyl halide such as methyl chloride. Yields reported are almost quantitative, even though the carbon atom efficiency falls to 63% when calculated from propylene, mainly due to low molecular weight alcohol being released during the reaction.

### 2.2. Emerging Technologies

Shifting from petrochemical feedstocks to renewable resources can help address some of the environmental concerns and make PAM production even more sustainable. Therefore, there is a growing interest in selective methods for transforming abundant non-food renewable feedstocks into monomers suitable for polymer production.

During the last decade, many routes using biobased raw materials have been developed. The first one corresponds to the valorization of glycerol byproduct obtained during the methanolysis of triglycerides extracted from vegetable oil. The worldwide annual production of glycerol is in the order of multi millions of tons, indicating a widespread availability. Glycerol can be converted into acrylic acid via various routes, but the simplest and most suitable is glycerol dehydration to acrolein in presence of a zirconium-based catalyst and oxygen [27]. The acrolein obtained is further oxidized to acrylic acid with the classical oxidation process previously described. Starting from pure glycerol leads to full conversion rates and a high selectivity, even though the catalyst deactivation using crude glycerol leads to poor economic performance. Furthermore, the alternative strategy, which consists of pre-purifying the glycerol, increases the overall costs of the process to uncompetitive levels.

Another alternative route has been largely explored, starting from dextrose being bioconverted with a genetically modified micro-organism strain to produce the intermediate 3-hydroxypropionic acid [28]. As a C3 building block, 3-hydroxypropionic acid offers potential in a variety of chemical conversions such as acrylamide or acrylic acid. Acrylamide is obtained by amidation of 3-hydroxypropionic acid with ammonia (gas or aqueous solution), and the 3-hydroxypropioamide thus obtained is dehydrated under high temperature to produce acrylamide [29]. This 3-hydroxypropionic acid building block can be directly dehydrated under a gas phase reaction at high temperature and under the presence of a catalyst to directly generate acrylic acid [30]. To give an idea of the cost of such a process, the University of Pennsylvania has estimated the fixed capital investment to be USD 266 M for an installed capacity of 160 kt per annum.

An alternative pathway to acrylic acid synthesis has been explored with a direct fermentation route starting from glucose. 3-hydroxypropionic acid is obtained as a key intermediate followed by three enzymatic steps, including CoA attachment to 3-hydroxypropionic acid by a CoA-transferase enzyme, dehydration of 3-HP-Co1 to acryloyl-CoA and detachment of CoA from acryloyl-CoA by a CoA-hydrolase [31,32]. However, this route suffers from very low productivity with a resulting concentration of acrylic acid as low as 0.12 g·L^−1^ within a 15-h timeframe. Moving the hydroxy leaving group from the beta position to alpha forms lactic acid, the latter being a structural isomer of 3-hydroxypropionic acid. This intermediate is currently obtained by bioconversion of lactoserum by the *Lactobacillus casei* strain. Lactoserum is highly rich in lactose and today is a byproduct of the milk industry. Production of lactic acid is more than 300,000 tons per annum and thus represents a potential viable precursor to acrylic acid. The main challenge with this structural isomer is the dehydration step, which is conducted at very high temperature and generates too many byproducts and side reactions, such as decarboxylation. This dehydration step is generally conducted using a catalyst, such as bulk salts, supported salts and zeolites.

Recently, a novel route has been patented to convert lactic acid, or its dimer lactide, to acrylic acid by dehydration using a novel catalyst composed of ionic liquid as a solvent, under moderate temperature, compared to conventional lactic acid dehydration processes [33], from 120 to 220 °C [34]. The catalyst is composed of tetrabutylphosphonium bromide and pyrophosphoric acid and thus allows for reaching a selectivity of more than 85%.

The last approach described in this section to access acrylic acid consists of the synthesis of poly (3-hydroxypropionate), which is an inert solid, chemically stable and easily transportable. The latter can be thermolyzed onsite to acrylic acid without any byproduct formation. Poly (3-hydroxypropionate) is obtained by polycondensation of beta-propiolactone, obtained by carbonylation of ethylene oxide with carbon monoxide in presence of an organometallic catalyst. This route is biobased, as ethylene oxide can be derivatized from bio-ethanol and CO being reduced from CO_2_, the latter being obtained by newly developed CCS technology (Carbon Capture and Storage), allowing this route to be circular [35,36]. Marginally, alternate routes using cinnamic acid, fumaric acid and muconic acid, three beta-substituted acrylics derivatives, can also produce acrylic acid by metathesis with ethylene [37,38,39].

Biobased acrylic acid has been extensively studied and described during the last decade, but, surprisingly, alternative bio-routes to acquire access to a greener acrylonitrile have been only developed by one stakeholder. The approach consists of converting glycerol to acrolein by dehydration. The latter is further reacted with ammonia and air by an ammoxidation process to produce acrylonitrile. The starting glycerol can be either obtained by hydrolysis of natural triglycerides from vegetable oils or bioconverted from sugars. The former fossil-based route (the so-called Sohio process) consists of the reaction of propylene with ammonia and air in vapor phase in presence of an organometallic catalyst. The overall yield reported is around 80% [40].

2-(Dimethylamino) ethyl acrylate can be obtained by a biocatalytic method, using the acryloyl-CoA as a starting material. A choline acetyltransferase enzyme catalyzes the esterification of the acryloyl-CoA with the dimethylaminoethanol, or choline, but the corresponding patent does not report molar yield. Former acryloyl-CoA is obtained with a 35% yield [41] by reacting acrylic acid with coenzyme A in the presence of ATP and a biocatalyst with S-acetyl coenzyme A synthetase activity.

Hydrolases are known enzymes capable of promoting a transesterification reaction, or ester hydrolysis, for various simple or complex molecules or for the isolation of a specific enantiomer using a racemic resolution technic [42]. More specifically, some papers report the direct esterification of acrylic acid, or transesterification of acrylic esters, using lipase B from Candida Antarctica. Various substrates have been studied, such as aliphatic alcohol [43], aryl ketone [44], diols [45], propargyl alcohol [46], alcohol bearing urethane moieties [47], polyols [48], 4-hydroxymethyl benzophenone [49] or alcohol bearing epoxy moieties [50]. One patent claims the specific case of obtaining 2-(Dimethylamino) ethyl acrylate by transesterification of acrylic ester with dimethylaminoethanol using a potential list of enzyme candidates such as lipase, protease or trypsin [51].

### 2.3. Circular Economy and Biobased Mass Balance Approach

Achieving a complete paradigm shift on key monomers production used by the PAM industry from fossil-based to biobased raw materials is not possible in a short period since a complete modification of the current production assets would be needed. This is a luxury the industry cannot afford alone given the level of investment involved. In addition, it will involve scraping the current production assets and, therefore, create more waste to be treated, without considering all the materials required to build new production plants.

Apart from the biobased origin of raw materials used by the chemical industry, a circular economy can possibly be discussed and implemented. However, to achieve a circular economy, stakeholders must find ways of collecting, circulating and processing the materials to turn waste into valuable feedstock. This concept is intuitive for some plastics, glass or steel, where the collection and reprocessing are established after being developed over many years. However, for chemicals, this is completely different, as they are commonly present in small quantities as additives to other materials or being used at very low concentrations (g·kg^−1^) in a complex matrix. Extracting and isolating such small quantities makes no sense due to the energy and resources required to collect and separate the chemicals from the after-use stream, likely turning this option into a less sustainable one compared to the use of fossil-based chemicals.

A mass balance approach (also called a non-segregated approach) is an important part of the solution and a necessary strategy towards more sustainable chemical production compared to the segregated approach described in the previous section on emerging technologies.

The mass balance concept enables a smooth and stepwise transition from fossil-based to bio-sourced or recycled resources by using the current production assets without the need to build expensive new production plants. The concept is well explained in a white paper written and released by the members of the Ellen MacArthur Foundation [52]. The mass balance concept has been used with success for many years in the biofuel and electricity industries. Applied to the PAM industry, the concept of mass balance consists of mixing fossil and renewable resources in the existing production assets while keeping a track record of the quantities used and allocating them to specific products. A chain of custody has been designed to create trust and transparency throughout the value chain regarding the quality and origin of chemicals while ensuring an appropriate allocation of this resource to finished goods using a conversion factor.

A third-party institute certifies the renewable content in the end-product following an auditable bookkeeping process. Several certification systems exist and, without being exhaustive, we can list RedCert^2^ [53], RSB [54], RSPO [55], Fair Trade [56] and ISCC+ [57], the latter being the most appropriate and widely used by the chemical industry.

With this system, renewable resources can either be biobased or recycled. The biomass is generally a second-generation waste being used as a feedstock for production of building block or platform chemicals. For example, the wood biorefineries generating pulp produce tall oil as a byproduct. Plastics, elastomers or even tires are today easily collected, and can be mechanically recycled. However, they can be fragmented to a liquid mix of hydrocarbon in a pyrolysis process and thus produce pyrolysis oil.

Both tall oil and pyrolysis oil are eligible feedstock, to replace fossil-based naphtha, for steam cracker production of propylene, which is the key building block of the PAM industry, thus creating a versatile loop of chemical value creation and smoothly switching the resources to a more sustainable and circular economy [58]. As an alternative source, bio-naphtha can be obtained by processing natural fats and oils or even used cooking oils [59].

In conclusion, it appears that some improvements have been made, especially regarding the production of acrylamide using an enzymatic route, reducing the environmental footprint and generation of waste. For other monomers, converting all the production from fossil-based raw materials to bio-sourced seems difficult since the current facilities would have to be replaced and adapted to the different manufacturing processes. As of now, the most plausible option is considering a mix of renewable and fossil feedstocks, considering the mass balance concept detailed above and working on other additional options through joint research programs. In the next section, we will focus on the PAM manufacturing process itself.

## 3. Sustainable Manufacturing Processes and Polymers

At the industrial scale, water soluble polymers containing acrylamide are mainly synthesized by a free radical polymerization process [60]. This multistep process mainly involves initiation, propagation and termination steps and is widely described in literature [60]. However, other types of radical polymerization processes can be industrially scaled up [61]. Among them, the reversible addition–fragmentation chain transfer polymerization (RAFT), the nitroxide-mediated polymerization (NMP) and the atom transfer radical polymerization (ATRP) can be cited [62]. Although these methods allow efficient control of the macromolecular structures, they represent only a small industrial scale volume when compared to free radical polymerization.

This section intends to discuss the polymerization processes of PAM in respect to the “green chemistry” principles.

### 3.1. Radical (Co)Polymerization of Acrylamide

Polymerization of acrylamide monomer is performed in water and can be conducted either in a homogeneous or a heterogeneous medium. The type of process is directly related to the desired macromolecular structures. Indeed, according to the Equation (1), the kinetic chain length, λ, the monomer concentration, [M], and the initiator concentration, [A], will affect the molecular weight [63].

(1) $$\lambda =\frac{k_{p}\;\left[M\right]}{2\;\sqrt{fk_{d}\left[A\right]k_{t}}}$$

where f is the initiator functionality and k_d_, k_p_ and k_t_ represent the dissociation, propagation and termination constant, respectively.

Thus, to achieve a desired average molecular weight, the manufacturer has to work at proper monomer concentration and adapt the process (temperature, initiator, etc.). For low to medium molecular weight polymers (10^3^–10^6^ g·mol^−1^), the process uses water as a solvent. The gel process is preferred to achieve higher molecular weights (up to 20 × 10^6^ g·mol^−1^). A heterogeneous medium, such as inverse emulsion polymerization, will yield polymers with properties between those obtained from liquid and gel processes. It is also the most suitable for branched and crosslinked polymers due to viscosity and swelling limitations.

Acrylamide is a convenient water-soluble monomer. Water is often considered a solvent of choice when considering PAM synthesis. Indeed, by referring to the “green chemistry” principles, it is the least hazardous solvent and, de facto, water-soluble polymers meet an important “green chemistry” criterion. Moreover, with its specific heat capacity of 4.180 kJ·kg^−1^·K^−1^ at 25 °C [64], water is among the best thermal sinks among the common fluids.

Usually, the monomer concentration is tuned based on the monomer reactivities. Indeed, monomers can be classified as more active monomers (MAMs), such as acrylamide or acrylates, and less active monomers (LAMs), such as *N*-vinylpyrrolidone or methacrylate. When considering acrylamide polymerization, the large amount of heat released (82.8 kJ·mol^−1^) results in a fast temperature rise [65]. This requires cautiousness to control the process. The concentration of acrylamide in the reactor will depend on the initiation method. Indeed, the use of thermal initiators at high temperature will require a decrease in the concentration of the monomer in a batch process. It is also possible to apply a semi-batch process using addition of monomer to control the instantaneous concentration of monomer in the medium and the heat released. In the case of a redox initiation, the initial temperature can be reduced to 0 °C, allowing us to work at higher acrylamide concentration. It is noteworthy to mention that the radical polymerization process yield is 100%, meaning that every molecule (monomers, additives and initiators) that goes in goes out in a form of a macromolecule. This also fits with the “green chemistry” principles. A life cycle assessment (LCA) performed in our group has shown that the raw materials are the most CO_2_ emitting components in the whole process. The transportation can be neglected when analyzing raw materials’ emission factors. The following paragraph will focus on the processes themselves.

### 3.2. Homogeneous Water Polymerization

Usually, the polymerization is carried out at a monomer concentration of 30% or less. The reason is that most polymerizations are initiated by thermal scission of an azoic or peroxide molecule and require a starting temperature of 40 °C to 80 °C. Apart from the raw materials used, the heating at the beginning of the process is the most CO_2_ emitting step, as water is heated using steam generated by a natural gas boiler. However, it is possible to start the polymerization at room temperature using a redox initiation system that will start the polymerization, allowing us to obtain higher molecular weight macromolecules while saving initial heating, thus reducing the overall CO_2_ emission. The final polymer is at a low concentration in water, and it should be supposed that transportation will support the excess of water in its CO_2_ balance sheet.

### 3.3. Gel Polymerization

The gel polymerization process is used to produce polymers in powder forms. It is a batch process that allows us to obtain high molecular weight polymer with a high purity. Monomers and additives are mixed and cooled down to 0 °C before initiating the polymerization by the addition of redox initiation system [66]. There is no mechanical stirring in the reactor. Once the polymerization starts, temperature rises, and no cooling is performed. It is an adiabatic process where the heat by the exotherm of polymerization maintains the monomers’ conversion until it reaches 100%. Once the polymerization is finished, the gel obtained passes through a granulator, a dryer and a grinder to obtain the desired particle size. Drying is continuous and is made with forced heated air, the latter being generated by natural gas combustion. This step is also an important CO_2_ emitting one in the process. It is noteworthy to mention that many initiatives related to possible improvements of this step can be considered. The first alternative is to source biomethane generated from fermentation of second generation agricultural waste or methanization of wastewater sludge. On other hand, one can mention the Qpinch chemical heat pump based on phosphates allowing the recovery of up to 50% of heat [67]. A powder with about 90% of active matter is obtained at the end of the process, allowing the transportation of a highly concentrated product and therefore minimizing the CO_2_ emissions compared to liquid forms, for instance.

### 3.4. Heterogeneous Inverse Emulsion Polymerization

The inverse emulsion (co)polymerization based on acrylamide [68] consists of the dispersion of water droplets containing the monomers into an oil phase with the help of surfactants. The monomer concentration can be increased up to 40% due to a redox initiation at room temperature, permitted by the presence of a water thermal sink allowing heat dissipation. In fact, the droplets are often considered independent microreactors where the polymerization takes place [69]. It is the less energy-intensive process among those presented in this review, since no heating is necessary. The process can add a subsequent step of distillation of the water and oil to increase the polymer concentration up to 65%. This CO_2_ emitting step can partially be compensated considering the oil recycling into the subsequent polymerization batch, thus making the oil circular and reducing the overall emission factor.

## 4. Reduction of Water Consumption and CO_2_ Emissions: Considering end User Benefits

### 4.1. Introduction: Handprint

The concept of total environmental footprint was introduced by Čuček et al. [70], considering the burdening (direct footprint) and the unburdening (indirect footprint) of the environment. The unburdening effects are beneficial, and they took as an example the replacement of fossil fuels by renewable fuels. This idea of indirect unburdening effect of a product has been developed by scientists, such as Kravanja and Čuček [71] when they developed a relative total sustainability index to assess social, economic and environmental impacts. However, the communication about beneficial impacts on the environment remained a delicate issue to avoid greenwashing, as shown by research made by Cronin et al. [72].

Even though the term handprint was at first presented by the UNESCO in 2007 as a measure of Education for Sustainability Development action [73], the chemical companies began to consider this expression as a contrary of footprint.

In 2013, the chemical sector introduced industry-related guidance (ICCA and WBCSD) [74] to calculate and report avoided emissions. Albeit the benefit of a product is a well-known concept, the handprint remains underused due to an unawareness of its basis and methodology. Grönman et al. [75] published a paper to provide a universal definition to the handprint concept and general guidelines for LCA-based quantification of carbon handprint. Handprint is defined as “the beneficial environmental impacts that organizations can achieve and communicate by providing products that reduce the footprints of customers” and carbon handprint as “the reduction of the carbon footprint of a customer or customers”. More specifically, it refers to the assessment of the environmental benefit due to the end use of a product. It is related to all resources that can be saved (water or energy) and the carbon emissions avoided. In 2018, the same authors developed an approach to calculate the reduction of the carbon emissions allowed by a product during its use. This approach is closely based on the life cycle assessment standards (ISO 14040, 2006; ISO 14044, 2006; ISO 14067, 2018) used to determine the carbon footprint of a product [76,77,78].

Moreover, the carbon handprint gathers growing interest, as it is in a close relationship with the carbon neutrality goals targeted by companies worldwide. Thus, having a high handprint could mitigate the carbon footprint and pave the way for a cleaner and more sustainable environmental profile.

Although the method to evaluate them is similar, the carbon footprint and handprint must be differentiated. The carbon footprint refers to the negative environmental impact due to the production (raw materials, processing or transportation), whereas the handprint refers to the positive impacts of the product during its use. Thus, the end user is looking for a product that increases its handprint while decreasing its overall footprint.

PAMs are used in several fields where a handprint can be evaluated. In addition to their primary function as flocculating agents in water treatment, rheology modifiers in Enhanced Oil Recovery (EOR) or water absorbing capacities in the case of superabsorbent polymer (SAP), these synthetic polymers contribute to reducing the overall CO_2_ emissions and water consumption.

### 4.2. Water Treatment

Wastewater treatment plants are among the major energy consumers in the cities because of their electricity consumption, which is higher than any municipal facilities (street lighting, schools, hospitals, etc.) [79]. This represents about 1% to 3% of the total electric needs of a country [80]. Wastewater contains very fine suspended solids, such as metals and organic or inorganic particles, that need to be eliminated [81]. Diverse technologies have been used to remove these particles, such as membrane filtration, ion exchange, flotation, precipitation, coagulation, flocculation and electrolytic methods [82]. The use of a flocculant proved to be an efficient method that has been extensively used for the treatment of various types of wastewater [83,84,85] and is among the most used separation method [86]. Coagulants (metal salts such as aluminum sulfate and ferric chloride) can also be used alone instead of flocculants [85,87], but it is less common due to the inefficiency at small dosage [81]. Polymeric flocculants, high molecular weight polymers, are efficient at low dosage and very popular in wastewater treatment [88]. For sludge treatments, consumption of a cationic polyacrylamide of 5.4 g·kg^−1^ of total solids is found to be the best economical solution [89].

Several studies were made, following life cycle analyses, to evaluate energy consumption of wastewater treatment (e.g., Tillman et al. [90], Hospido et al. [91] and Wenzel et al. [92]). They show that the most important environmental effect is due to electrical and thermal energy consumption. Another LCA was made by Remy et al., in 2013 [93] considering all relevant processes of sludge treatment and disposal. It explains that sludges coming from sedimentation are called primary sludges and have a total solid concentration of 5%, whereas sludges coming from the activated sludge process are called excess sludges and have a total solid concentration of 1.2%. Excess sludges are thickened and then mixed with primary sludges to be fed to the digestors. Sludges are digested and dewatered by centrifuges with polymer addition to increase its total solid concentration from 4% to 27%. Dewatered sludge is then incinerated and the heat recovered into energy. Environmental benefits because of the use of polymers for dewatering can be evaluated due to a comparison between a theorical case without polymer use and a practical case with polymer.

For instance, the authors worked on a case study where the following assumption was made without dewatering polymer and the same process is used to treat the sludge, but, in return, it is necessary to evaporate additional water with natural gas to balance the higher water content of the sludge to reach the wanted total solid concentration, which corresponds to the concentration normally obtained with polymer. Emissions due to this water evaporation process are clearly avoided with the use of polymer.

Emission factors of gas (0.230 kg CO_2_e·kW^−1^·h^−1^) and electricity (0.0599 kg CO_2_e·kW^−1^·h^−1^ in France in 2020) come from the ADEME Database [94]. An emission factor of 3.25 kg CO_2_e·kg^−1^ is considered for the cationic polymer made with fossil raw materials. With the use of sustainable certified raw materials, it is possible to use a polymer with a lower emission factor. By using bio-sourced or recycled raw materials (segregated or not, such as with the ISCC+ mass balance approach), the emission factor of a cationic powder can decrease to 0.60 kg CO_2_e·kg^−1^. The assumption of an agitation for 1 h (overestimated) with two motors (power = 3 kW) to dissolve polymer and add it to the sludge is also made.

Table 1 shows that polymers allow a decrease of 87% in CO_2_ emissions. This decrease can reach 97% if raw materials used to manufacture the polymer are ISCC+ certified. Polymers also allow the recovery of clean water, without suspended particles. This example clearly shows that the handprint largely exceeds the footprint and performance is the lead criterion in this process from an environmental standpoint.

### 4.3. Agriculture

**Figure 1:**
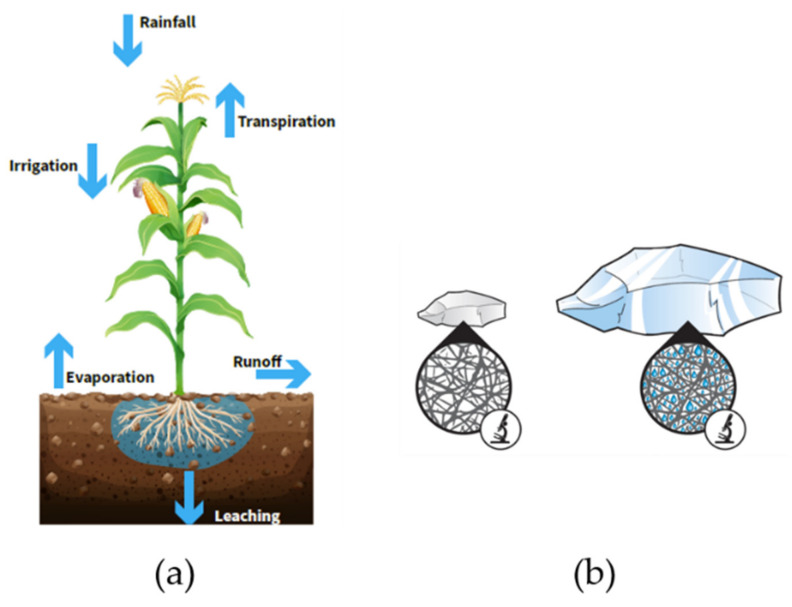
(**a**) Mechanisms of agricultural PAM uses and (**b**) SAP structure (left dried; right swelled).

PAMs have been used for more than three decades as soil conditioning or water retention agents [95]. These two areas are distinguished by the nature of the polymer used. Indeed, in soil conditioning [96], the polymer has linear structure and is assimilated as a flocculant, whereas the water retention polymer is a crosslinked anionic network with superabsorbent capabilities [97]. All these polymers are designed to avoid water loss (evaporation, run off or leaching) and increase their retention and avoid hydric stress, thus improving crops yield (Figure 1a).

The use of PAMs as soil conditioners started in the 1950s with the evaluation of the Krilium from Monsanto Chemical Company [95]. However, due to high price and high usage concentration in the fields, the project was cancelled. It was only in the 1980s that the idea of PAM soil conditioners was resurrected due to a decrease in the acrylamide’s price and advances in polymer science. Wallace Laboratories intensified the evaluation of these polymers in the agricultural field [95]. Since then, PAMs found various applications to enhance soil quality and water usage efficiency, give better efficiency of fertilizer use and increase crops yield.

PAMs as soil conditioners are similar to a flocculant used in wastewater treatment. The structure of such polymer is linear, bears anionic charges and exhibits a high molecular weight of about 10 × 10^6^ g·mol^−1^. Due to its flocculation properties, the polymer enhances the cohesive attraction between fine particulates, thus creating a better structuration of the soil and a controlled water infiltration while reducing its erosion. The water distribution in the soil is achieved by the fact that the small particulates are adsorbed onto the polymer and cannot plug the soil pores anymore. Thus, water can diffuse in the soil to irrigate the roots, contributing to the water’s efficiency and its reduced consumption.

For instance, Stern et al. found that the use of PAMs increased crops yield due to improved water distribution through its infiltration and greater water use efficiency [98]. In the same way, the pretreatment of soil by PAM reduced the runoff and the soil loss by 70% and 75%, respectively, compared to non-treated soil [99].

To summarize, PAMs have shown great properties in erosion prevention and leaching control by stabilizing the soil surface structures and increasing water penetration by avoiding pore plugging [100]. All this actively contributes to water savings by reducing water irrigation needs while enhancing crops yield, whichever irrigation method is used (furrow, sprinkler, drip, flood, or rainfall).

The question of anionic PAM toxicity in agriculture was raised. In 2009, Weston et al. confirmed the non-toxicity of PAMs used as long as they were synthesized in a non-oil-based manner [101]. Indeed, when oil-based inverse emulsion polymers were used, a slight toxicity was observed due to the presence of mineral oil. However, when a non-oil-based PAM was used, no toxicity was observed, even at concentrations ten times higher than those usually used by the wastewater industry, proving the innocuity of the PAMs.

Water retention polymers are three-dimensional chemically crosslinked networks and bear a high anionic charge density [102]. They are superabsorbent polymers and are insoluble in water but water swellable (Figure 1b). They can absorb about 200 to 1000 times their weight in water, which represents a great interest in agriculture, especially where water scarcity is a major problem. Therefore, they are complementary to the soil conditioners. Synthetic polymers are preferred over natural ones due to their better water retention capacities, low cost, availability and durability [97].

**Figure 2:**
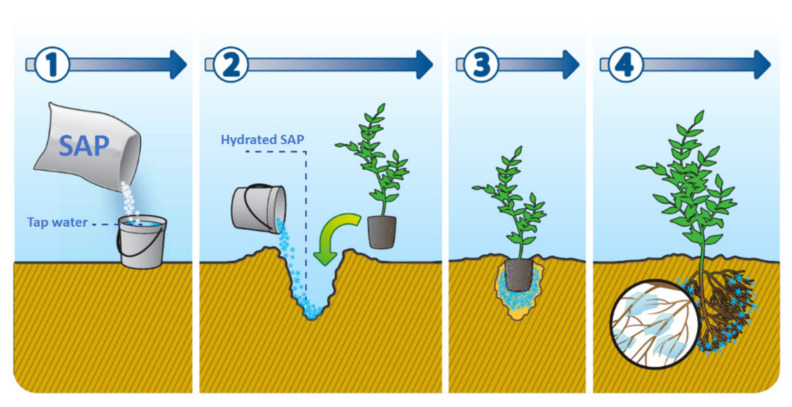
Use of SAP in agriculture.

The water absorption and retaining capacities of these polymers help to reduce any water stress for the plant [103]. This contributes to water savings in that water can remain in the soil in the proximity of the seeds and roots, keeping moisture at a sufficient level without the need of additional irrigation (Figure 2).

For instance, the use of SAP in oat crops proved to be efficient especially under hydric stress [104]. The authors also proved that the use of SAP reduced the activity of antioxidant enzymes responsible for reducing the plant capabilities to fix carbon under drought conditions and also its growth.

Besides water absorbing properties, it has also been demonstrated that SAP could be used for the slow release of fertilizers, enhancing productivity and reducing the amounts needed, thus decreasing their impact on the environment [105].

Although the general trend is highly biodegradable polymers, the authors must point out that such polymers would be less effective in the agricultural field, where the polymers must be stable enough to swell and release water over long periods of time. Therefore, it is needed to find a way to synthesize polymers exhibiting good swelling capacities which are not persistent in the environment.

It should also be emphasized that, depending on the crosslinking density, some SAPs may fall into the definition of microplastics proposed by the European Chemical Agency (ECHA). In opposition to linear PAMs, which exhibit a water solubility greater than 2 g·L^−1^, SAPs may be impacted by a restriction if a specific regulation is approved by ECHA. The final dispositions will be known by 2022 [106,107].

### 4.4. Improved Oil and Gas Recovery

The extraction of oil and gas is facing a considerable challenge with the goal of net-zero emission by 2050 and the unprecedented crisis due to COVID-19. Indeed, because of the pandemic, oil demand and prices decreased, leading to a production reduction. Returning to a pre-COVID-19 production level demands more efficient and faster ways of production [108]. Currently, water injection is the most used oil recovery technique for conventional reservoirs and allows us to reach recovery factors in the range of 20% to 40% of the original oil-in-place with a large amount of contaminated wastewater (up to 90% of the total produced liquids) [108].

Methods were developed to improve oil recovery efficiency, among which is polymer flooding. It consists of the addition of polymers to water in order to increase its viscosity and consequently the amount of recovered oil [109]. It improves the oil sweep efficiency within the reservoir, leading to a decrease in water consumption and a decrease in CO_2_ emissions [110]. Several projects of polymer flooding have been documented [111,112,113], revealing a significant water cut reduction in a short time and an improvement in oil production. Water-to-oil ratio can be typically reduced by a factor up to six. It can be a solution to stop the oil decline [114]. Various type of polymers can be used for polymer flooding, but polyacrylamides are the most used in chemical-enhanced oil recovery projects [115].

Morice et al. studied the use of polyacrylamide to reduce carbon intensity and to increase oil recovery [108]. They benchmarked the energy consumption related to oil recovery between water and polymer injection. They included water treatment, polymer synthesis (contribution of raw materials, energy for polymerization and conditioning), transportation, polymer injection, pumps, artificial lift, fluid separation, heating and oilfield chemicals consumption. They demonstrated that the use of polyacrylamides allows, for each barrel of oil produced, a reduction of three to six times the CO_2_ emissions, a reduction in injected water by 60% to 70% and a reduction in produced water by 70% to 80%. Reduction in oilfield chemicals consumption compensates emissions due to polymer use, as their manufacturing process is less efficient than that of polyacrylamides. As a fraction of polymer stays in water, re-injection of water leads to a reduction in fresh polymer consumption by up to 35%.

Efficiency of polymers can be evaluated according to various points of view. It would take 10 years with waterflooding to recover the same amount of produced oil that is recovered in 2.6 years with polymer flooding, the latter emitting 5600 mT less CO_2_. Compared with iso-CO_2_ emissions (baseline reference is CO_2_ emissions after 10 years of waterflooding), oil recovery increases (+190% with polymer flooding) while recovery time decreases from 10 to 8.8 years.

Farajzadeh et al. also evaluated benefits due to polymer injection [115]. In their example, a decrease of two to five times in CO_2_ intensity per barrel of oil compared to waterflooding is observed.

Water is also used in stimulation processes to create more and larger flow paths to recover gas or oil from shale rocks (so-called shale gas or shale oil). To achieve high rates, it is necessary to decrease the friction losses during fluid pumping along several kilometers from the surface to the shale formation. This is possible due to the addition of polyacrylamides, which dampen the flow turbulences and help reduce the energy required to displace large volumes of water at high pressures, thereby decreasing by a factor of two or more the fuel needed to power the pumps and trucks.

In conclusion for this section, it appears that polyacrylamides have a huge impact on the reduction in environmental footprint or on the opposite handprint, CO_2_ emissions and water consumption for all the applications considered: wastewater treatment, agriculture and even the extraction of resources. In the last section, we will discuss what occurs once the polymer has been used and its fate in the environment.

## 5. Environmental Fate of Polymers

### 5.1. Current Framework and Procedures for the Evaluation of Polymer Biodegradation and Challenges

To study and understand the toxicity of chemical material, which is essential to improve and predict environmental impact, the fourth section (Health Effect) of the OECD Guideline for Testing of Chemicals was developed. Water-soluble polymers have been studied for a long time regarding toxicity, the most used worldwide being the polyacrylamide. McCollister, in 1965, established the safety of polyacrylamides in the public health aspect for many applications wherein small amounts may possibly occur in the food or drink of animals or human subjects [116]. Those results were confirmed recently by Hensen et al. [117] and Farkas et al. [118] on marine fish species. Other studies have been published to highlight the difference of toxicity between acrylamide—the building block of polyacrylamide—and polyacrylamide [119,120]. Indeed, King et al. and Smith et al. confirmed the innocuity of polyacrylamide, whereas acrylamide monomer is classified as neurotoxic. In another study led on polyacrylamide used in agriculture, Anh et al. showed that polyacrylamide does not depolymerize on toxic acrylamide monomer, despite a misleading title [121]. This result was confirmed by Vers through a high-performance liquid chromatography method [122]. Vers’s study confirmed that the polymer does not degrade to acrylamide in the presence of glyphosate or sunlight or any combination of the two. Reber et al., in 2007, thermodynamically proved that polyacrylamide cannot degrade to its starting monomer (acrylamide), as the energy needed could not be reached in environmental conditions [123].

With people being more and more concerned with what daily products are composed of, certifications highlighting raw material origins emerged. For example, in the home and personal care field, Cosmos, Ecolabel, Nature and others came up. To control information given to the customer, two standards leading to origin of product have been published: ISO 16128-1:2016 [124] for the definition of natural and organic cosmetic ingredients and ISO 1628-2:2017 [125] describing approaches to calculate natural index. Other certifications, such as NSF international, Food Contact, BFR and CEFAS are today used to label chemical products or compositions. Recently, certifications such as ISCC+/RedCert^2^/RSB have been developed to improve the biomass balance approach and improve the sustainability of chemicals.

Simple tests for the measurement of biodegradability of a given chemical substance have been designed and adapted for operation in the laboratory and are available as normalized tests. These tests are widely used to assess the biodegradability of a given chemical substance and are more adapted for small chemical species, soluble in water and not volatile. With these features, the chemical substance can be dissolved in a water-based medium, also used as a culture medium for the micro-organisms and made available as the sole carbon source. After utilization of the carbon source by the micro-organisms, the end products can be analyzed over time to monitor their evolution and calculate the biodegradation rate. In this case, the process of biodegradation is simply explained: the chemical substance is expected to rapidly cross the biological barriers of the micro-organisms, provided its size is small enough [126]. Being internalized, it will be subjected to degradation by the enzymatic machinery of the microbial cell, similar to any carbon source naturally metabolized by the organism. Upon complete mineralization, under aerobic conditions, all the carbon of the chemical substance will be converted into carbon dioxide or incorporated into biomass. Normalized biodegradation tests, based on these general principles, are the widely adopted tests proposed by the OECD (Organization for Economic Co-operation and Development). These tests can be divided into three categories: tests for ready biodegradability or screening tests (OECD 301 series), tests for inherent biodegradability (OECD 302 series) and simulation tests that are more adapted to mimic the conditions found in the environment in the laboratory (Table 2). The most used are OECD 301F (manometric respiratory test with Oxitop bottles), OECD 302 B (Zahn-Wellens test) and OECD 306 (Biodegradability in seawater).

Challenges associated with the application of these tests to a given substance are numerous. To cite only a few, complexity of the molecule (and the necessary fragmentation of a polymer), quantity and quality of the microbial inoculum (with the necessary presence of specific degraders), duration of the test (28 days is not realistic for polymer degradation), other abiotic environmental factors that can promote degradation (oxidation, hydrolysis and photodegradation) and finally, choice of the right analytical method, are key.

The shortcomings of the current normalized tests also lead us to introduce new promising tools, such as the BlueSens test system, analyzing the biodegradation from the viewpoint of carbon mass balances and related O_2_ consumption [127]. However, these tests still have to be recognized by the scientific community and the authorities.

### 5.2. State-of-the-Art and Discussion

Polyacrylamide biodegradation is a complex topic that has been reviewed in the literature [128,129,130,131,132,133,134]. When released into the environment, the polymer molecules will be exposed to various abiotic (i.e., physiochemical degradation) and biotic (i.e., degradation by the action of micro-organisms) conditions that are prone to change the molecule functionality and molecular mass. PAMs are sensitive to physical stresses and the action of micro-organisms; all those mechanisms will contribute to transform the molecules over time. When some conditions are met, micro-organisms are capable, through various pathways, of bio-transforming the molecules (i.e., all structural changes at molecular level), leading eventually to their mineralization (i.e., the use and transformation through different metabolic pathways of the polymer and its conversion to mineral species, including, for example, CO_2_). Different aerobic and anaerobic protocols, in various incubation mediums such as inoculated solutions, sludge, fresh and processed water or directly in soil, combined or not with pre-degradation steps, have been developed to understand and monitor the polymer structure changes over time and ultimately determines the fate of PAMs in the environment.

Physiochemical degradation has been studied mainly for its capability to promote polymer chain scission and has been observed, using various analytical technics, by applying shear, temperature, microwaves, UV and oxidation to polymers [135,136,137,138,139,140,141,142,143,144,145,146]. Concerning structural changes and biotransformation by micro-organisms only, it has been demonstrated that acrylamide moieties undergo a rather rapid deamination, leading to the formation of corresponding acrylate salt or acrylic acid and ammonia or corresponding salts [130,147,148]. Most cationic moieties of cationic PAMs are also prone to prompt ester hydrolysis, also resulting in the formation of corresponding acrylate salt or acrylic acid and the release of quaternary ammonium products. Those resulting quaternary ammonium species (mainly 2-Hydroxyethyl-trimethylammonium chloride, for most commercial products) have found to be biodegradable either in aerobic conditions or anaerobic conditions [137]. Those mechanisms are commonly accepted as the first step of polymer biotransformation. In aerobic and anaerobic conditions, they are promoted by extracellular enzymes, such as amidase for deamination. Polymer carbon-carbon chain scission by micro-organisms is more complex [149,150,151]. It is commonly accepted that most PAMs are too large to be able to pass microbial membranes and therefore for micro-organisms to be capable of efficiently biodegrading them [131,152]. Chain scission must first occur to lead to biodegradation, and this has been evidenced in various aerobic or anaerobic conditions and also promoted by the activity of cell-associated enzymes, either extracellular or located on the cytoplasmic membranes [130,153]. In aerobic conditions, experiments support that oxidation reactions on the carbon chain are catalyzed by oxygenase. The oxidation reaction is then responsible for carbon-carbon single bond breakages, leading to the main carbon chain backbone degradation toward smaller molecular fragments by a proposed mechanism of a cascade of reactions, starting somehow in a similar fashion to the α-oxidation of fatty acids. In anaerobic conditions, the mechanism of PAM degradation is proposed to happen first by hydrolysis into poly(acrylic acid) (PAA) by amidase, followed by the formation of acetic acid or other volatile fatty acids through the action of a cascade of enzymes, namely dehydrogenases, phosphotransacetylases and acetate kinases [148,154]. Broadly speaking, polymer molecular weight reduction is progressive and continuous and is usually observed over a time span of months [149].

Polyacrylamide copolymers, either anionic or cationic, are potentially quickly converted to corresponding polyacrylate salts by deamination or hydrolysis. Therefore, biodegradation of related polyacrylic acid (or related salts) provides insights in polyacrylamide biodegradation and their fate. More specifically, the study of small oligomeric PAA fragments provides a good model. A comprehensive report by the European Centre for Ecotoxicology and Toxicology of Chemicals compiled data showing that ^14^C labelled polyacrylates of mass ranging from 1000 to 10,000 g·mol^−1^ could be biodegraded when incubated in water-inoculated solution with activated sludge, river, soil or sewage sludge [155]. The extent of mineralization of PAA oligomers by micro-organisms have been reported from several studies to be from 8 to 80%, depending upon the oligomers’ molecular weight, type of micro-organisms and conditions on time span, ranging from days to months [133,156,157,158,159]. During biodegradation, formation of acrylic acid monomer is not reported, this substance being easily metabolized by micro-organisms to CO_2_ within days [158].

Several studies have clearly evidenced the slow molecular weight decrease in PAMs by micro-organisms in aerobic and anaerobic conditions [147,149,150,160,161,162,163,164,165,166,167]. This degradation can be concomitant or not, with a partial mineralization of the polymer when observed. A quite comprehensive three-year study assessed the degradation of a ^14^C labelled cationic PAM used to dewater municipal sludge which was then integrated into an agricultural top-soil [147]. Only carbon atoms on the main polymer backbone were tagged, and the biodegradation was followed by measurement of the radioactivity over time, considering that disappearance of radioactivity corresponds presumably to the emission of carbon dioxide as mineralization product. This is a rather conservative approach, as it is not considering other possible degradation pathways, but at the same time it provides a good understanding of the extent of the carbon-carbon backbone chain scission to mineralization. The polymer was found to be immobilized in the resulting soil matrix without migration or deleterious effects on plant growth or yield. No uptake of acrylamide by the plants or any other form of radioactivity was observed. The molecular weight of the polymer was drastically reduced when extracted from the soil matrix and was degraded biologically by at least 20% in two years, giving a degradation half-life of 5.4 years [147].

Other studies do not report strong evidence of acrylamide reformation during polymer degradation [166,168]. This is mainly explained by the fast deamination mechanisms, evidence of enzymes cleaving macromolecules randomly and statistically more occurrence of having a chain scission within the polymer chain rather than at the end units. PAMs are likely to be discharged in the environment as polymers soluble in water but due to their chemistry, they are quickly removed from water bodies through adsorption and flocculation of suspended solids within a few hundred meters of transport from their point of application [131].

The state-of-the-art concerning the fate of PAMs in the environment supports that PAMs could likely be moderately released to the environment in water run-off. Their pending functionalities are rather rapidly hydrolyzed to non-persistent products, whereas the decrease in molecular weight by the action of micro-organisms only is slow. Overall, biotic or abiotic mechanisms all play a role, and the degradation yields polymers of smaller molecular weight of the same chemical composition or with a tendency to evolve toward PAA oligomers. Acrylic acid or acrylamide reformation have not been evidenced. To the best of our knowledge, high molecular weight PAMs demonstrate biodegradation rates with half-lives of years. This would demonstrate that such polymers are non-persistent but slow to degrade without generating concerning or hazardous chemicals during the process.

However, the rate of biodegradation requires to be studied more comprehensively, as results may vary strongly given the conditions. For instance, the fate of PAM polymer in marine environments implies a first step of dilution in a large volume of seawater and different dynamics than in soil. A study performed by SINTEF has shown that PAMs remain dissolved in the water phase and are not be driven by sedimentation [169]. Kinetics could be slower because of the lower concentration of micro-organisms in this environment. The small concentrations of the polymer are not amenable to a direct measurement by conventional techniques, nor are the release of carbon dioxide for an exact evaluation of biodegradation. It is worth noticing that several of the normative standards used for legal, regulatory or certification frameworks do not seem to be appropriate to quantify the biodegradation of PAMs, which is slow and progressive, and are outside the commonly used 28 days to months range of biodegradation observation for the most popular OECD methods. In addition, holistic approaches with systematic monitoring of molecular weight or functionality changes over time using correct analytical methodologies combined with respirometry methods that will evidence without bias concomitant biodegradation (for example, CO_2_ monitoring using polymer ^13^C or ^14^C tagging) are lacking. Evolution of micro-organism colonies and monitoring of enzymatic activities during biodegradation should also be further studied [167]. Such framework will certainly provide better insights toward the understanding of PAMs’ fate in the environment as well as a path to innovation for designing molecules that may be prone to easier degradation. 

## 6. Macromolecular Engineering to Favor Biodegradability

As highlighted in the previous section, similar to most of the synthetic polymers made of vinyl monomers, polyacrylamide derivatives are resistant to degradation, which is a potential concern for some uses. This section deals with the different strategies available, based on macromolecular engineering, to improve the degradability of those polymers. Three main technological approaches have been identified to confer degradability to synthetic water-soluble polymers.

- Copolymerization with biodegradable moieties

As mentioned, most of the backbones of synthetic polymers made of vinyl monomers resist degradation, but there are some exceptions, such as poly (vinyl alcohol) [170]. The idea is to include degradable units (vinyl alcohol for instance) during the polymerization of vinyl monomers to induce some biodegradation properties in the copolymer.

- Blending natural and synthetic polymers

Blending, in the process, a natural biodegradable polymer, such as a polysaccharide with a synthetic polymer, is one way to bring biodegradability to the composite material. This can be achieved either by blending a biodegradable polymer such as polysaccharide with vinyl monomers during the polymerization stage (the final material is assimilated to interpenetrated network, IPN) or by combining polymers together through covalent bonds (grafting material). The latter option relies typically on using polysaccharide as a reducing agent able to initiate polymerization of vinyl monomers.

- Introducing cleavable units within the backbone of the polymer

The robustness towards degradation of synthetic polymers is mainly linked to the C-C backbone, as there is no possibility to hydrolyze or properly oxidize the chain in an easy manner, except for the specific case of poly (vinyl alcohol). The main mechanism of degradation is based on chain breaking through high shearing or oxidation through the end-chain groups. In this matter, high molecular weight chains are not favored. Therefore, breaking the polymer in small fragments by inclusion of weak links on the main backbone is one way to improve degradability. Introducing a small number of weak links is normally sufficient, since one link is enough to provide, on average, a reduction in the molecular weight by two. The introduction of labile units on the polymer backbone has been widely studied and many strategies have been proposed. For instance, most of the technologies are based on controlled radical polymerization technologies associated with specific chemical groups. Some of them have been applied to hydro soluble polymers. A more detailed description of those three macromolecular approaches is disclosed hereafter.

### 6.1. Copolymerization with Degradable Monomers

Poly (vinyl alcohol) (PVA) is recognized as one of few vinyl polymers that can have a high biodegradation rate [170]. This is possible due to the presence of hydroxyl groups, which condition hydrophilic nature of this material. In 1936, it was observed that PVA sustained ultimate biodegradation when submitted to the action of *Fusarium lini*, a phytopathogenic fungus, producing carbon dioxide and water as a result of extracellular attack by adehydratase enzyme [171]. Some papers in the literature mentioned the introduction of vinyl acetate unit in poly (sodium acrylate) to improve the biodegradability of the polyelectrolyte [172,173]. The use of degradable units seems to induce biodegradation of the copolymer if the bacterial strain is well selected. *Pseudomonas* species and *Brevibacterium incertum* confirmed the active participation of a hydrolase in the biodegradation mechanism [174].

### 6.2. Blend of Natural and Synthetic Polymers

Natural polymers, such as polysaccharides, hold an advantage over the synthetic polymers because of non-toxicity, biodegradability and easy availability. These kind of polymers have unique properties but limited scope of long-lasting material due to shelf life and bacterial attack [175]. Combining natural and synthetic polymers can be made through two different ways.

#### 6.2.1. Interpenetrated Network (IPN)

Linking a synthetic polymer chain covalently on a natural polymer (such as polysaccharide) is not the goal for creating an Interpenetrated Network (IPN). The empirical approach is to initiate polymerization of vinyl monomers in the presence of a natural polymer to create a network of entangled chains. Most of the time, a crosslinker (polyfunctional vinyl monomer) is introduced in the process to fix the internal structure of the network. By using acrylamide monomer and methylene-bis-acrylamide (MBA) as crosslinking agent, a gel with water-swelling properties is obtained (hydrogel). Many types of natural polymers have been described in the literature for the preparation of IPN [176,177]. The cost is one of the drivers of selection along with specific functional groups borne by the natural polymer. Starch, cellulose (and derivatives), natural gums and chitosan are the main polysaccharides associated with acrylamide type monomers. Many studies on hydrogels have been dedicated to medical applications [178,179].

#### 6.2.2. Grafting Material

**Figure 3:**
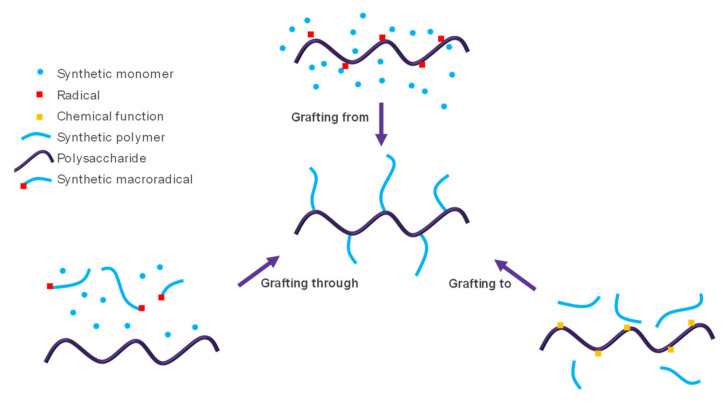
The three main mechanisms to prepare hybrid polymers.

Grafting of synthetic polymers onto natural polymers has invited great interest [180]. It is one of the easiest methods to keep the properties of synthetic polymers with improved biodegradability. For this purpose, three main mechanisms have been identified and depicted on Figure 3:

- “Grafting through”: Synthetic monomers are copolymerized with a natural polymer containing polymerizable groups [181].
- “Grafting from”: The natural polymer is used as a macroinitiator for the polymerization of synthetic monomers [182,183].
- “Grafting onto”: Functional natural and synthetic polymers are connected through covalent bonds [184].

**Figure 4:**
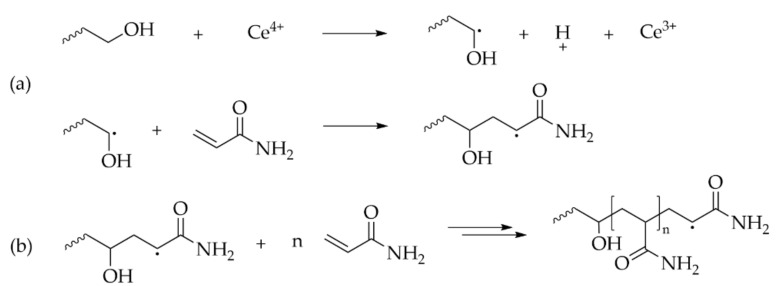
Mechanism of polymerization of acrylamide using Ce4+/alcohol redox system. (**a**) Radical generation and chain initiation; (**b**) propagation.

As radical polymerization is a useful method for the polymerization of a wide variety of vinyl monomers, the initiation through radicals located on the polysaccharide backbone (“Grafting from”) is favored. This polymerization can be obtained by different initiator systems, among them azo-bis-isobutyronitrile (AIBN), persulfate salts and peroxides. Ceric ammonium nitrate (CAN) is one of the most studied chemicals, as it can efficiently induce a radical on the polysaccharide which can initiate the polymerization of the vinyl monomer [185]. The mechanism of polymerization is described on Figure 4.

Besides polysaccharides, different degradable polyols (polylactides, polycaprolactone diol) have also been described in the literature as reducing agents [179].

### 6.3. Cleavable Polymers

#### 6.3.1. Disulfide

**Figure 5:**
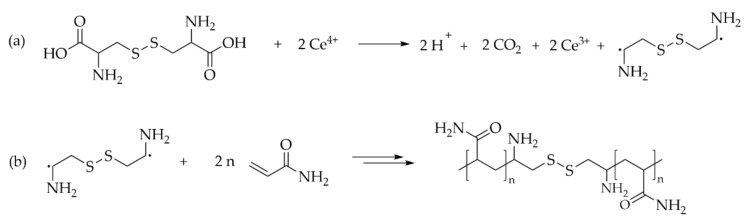
Mechanism of polymerization of acrylamide using Ce^4+^/Cystein redox system. (**a**) Radical generation and chain initiation; (**b**) Polymerization with acrylamide.

Disulfides are well-known chemical groups able to dissociate in thiol functions after undergoing a reduction reaction [186]. The introduction of disulfide groups in the main chain of synthetic vinyl polymers is one way to reduce the molecular weight after triggering. Cystine (disulfide form of the natural amino acid, cysteine) has been described as a potential weak link able to react with acrylamide [187]. This reaction is led by Cerium IV following the scheme described on Figure 5. The final polymer owns the degradable function in the middle of the chain, leading to a reduction in molecular weight by a factor of two.

#### 6.3.2. Polydisulfide

**Figure 6:**
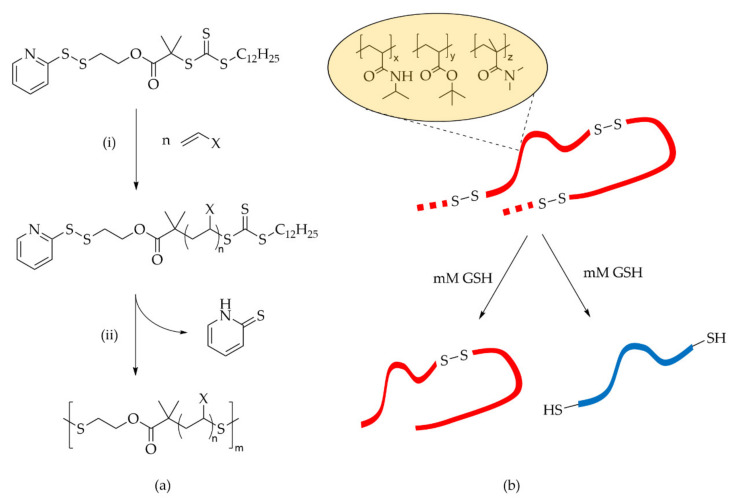
Scheme of (**a**) synthesis of polydisulfide and (**b**) degradation with glutathione.

To gain in efficiency with a disulfide approach, the idea is to include several S-S groups in the same polymer chain. This can be achieved through a RAFT-derived macromonomer [188]. The strategy of preparation of disulfide-linked polymers from RAFT-derived macromonomers requires aminolysis of the RAFT agent to yield a thiol-terminated polymer (Figure 6). The latter can react with neighboring chains, giving a polycondensation-type polymerization. The introduction of glutathione is one way to break the Sulfur-Sulfur bond and degrade the polymer. This may be applied to different hydrophilic monomers, such as *N*-isopropylacrylamide and *N*,*N*-dimethylacrylamide. The main issue of this process is to acquire access to the initial RAFT macromonomer.

#### 6.3.3. Azo-Bis

**Figure 7:**
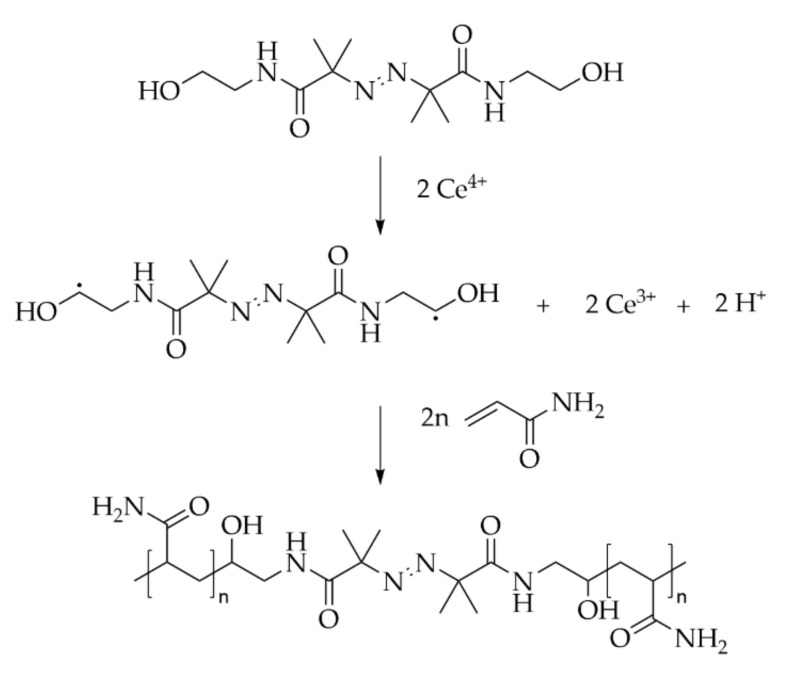
Mechanism of polymerization of acrylamide using Ce^4+^/VA-086 redox system.

A very interesting approach to the insertion of weak links in the main chain is the use of diazo chemicals [189]. Diazo are typically used as thermal initiators for radical polymerization. Including this chemical in the chain allows the degradation of the chain upon heating. A diazo chemical functionalized with alcohol groups is commercially available and known as VA-086 (2,2′-Azobis[2-methyl-*N*-(2-hydroxyethyl) propionamide]). The use of Cerium IV as oxidizer at low temperature allows the initiation of the polymerization of vinyl monomers on the alcohol part of the molecule (Figure 7).

#### 6.3.4. r-ROP

**Figure 8:**
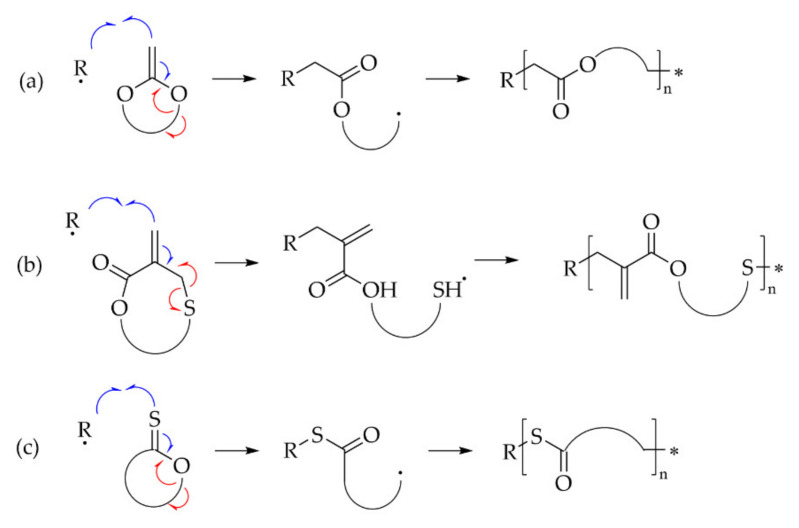
Radical ring-opening polymerization from (**a**) Cyclic Ketene Acetal, (**b**) Allyl sulfide lactones and (**c**) Thionolactones.

To achieve significant degradation of the polymer, the insertion of multiple main-chain degradable functionalities is mandatory. Radical ring-opening polymerization (r-ROP) of cyclic monomers remains the process of choice to achieve this goal [186,190,191,192,193,194]. The principle of this technology is to initiate a radical polymerization leading to the ring-opening of the monomer, thus allowing the radical to propagate on another monomer. The final polymer chain includes chemical groups which are subjected to further hydrolysis. This has been well described in the literature, and several research groups are still actively working on this process. Different types of monomers able to undergo an r-ROP process have been studied and are shown on Figure 8.

- Cyclic Ketene Acetal (CKA) is one of the most studied families of monomers [191,195,196,197,198]. Different monomers have been described in the literature and are now commonly used. The chemical function remaining in the main chain after polymerization is an ester.
- Allyl sulfide lactones are also described as monomers able to undergo an r-ROP process [199,200]. Here, again, the weak unit formed is an ester.
- Thionolactones are cyclic monomers used in a specific polymerization process designed as TARO (Thiocarbonyl Addition Ring Opening) [201,202,203]. Thioester groups are formed in the main chain.

#### 6.3.5. Self-Immolative Polymer

**Figure 9:**
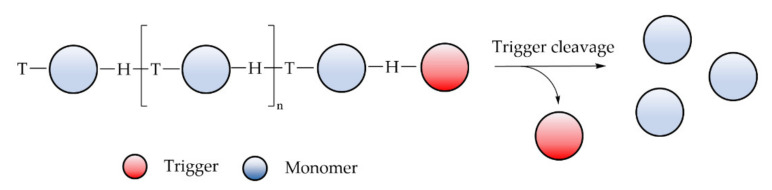
Scheme of disassembling of a self-immolative polymer.

A new field of research has been recently reported in the literature which deals with self-immolative polymers [204]. Those polymers are made of building blocks linked together through head-to-tail organization. The polymer is designed to sequentially disassemble into building blocks once the disassembly process is initiated by a triggering event (Figure 9).

**Figure 10:**
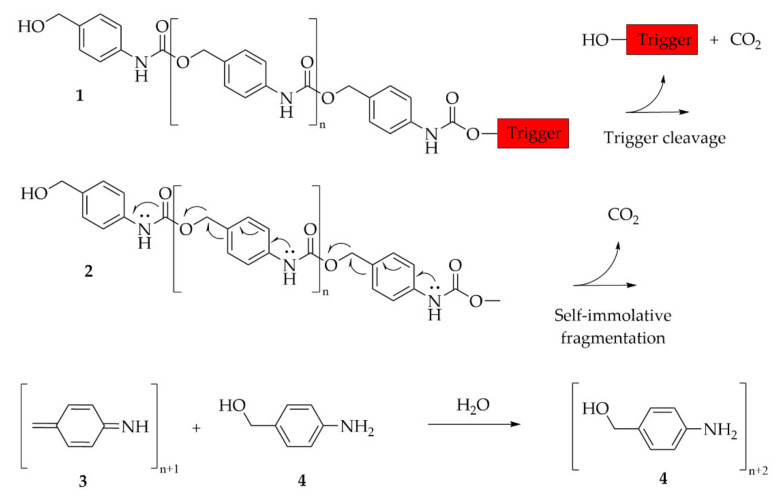
Description of the chemistry to disassemble a self-immolative polyurethane polymer.

This mechanism can be applied to polyurethane chains with a protective group on the amine that will act as a trigger. The disassemble reaction leaves amino alcohol units along with CO_2_ release (Figure 10). However, this very attractive mechanism is difficult to apply to polymers made of vinyl units. One can still imagine grafting vinyl polymer chains on building blocks.

### 6.4. Conclusion on Macromolecular Engineering

From a chemical point of view, one of the most interesting processes to obtain improved degradable polyacrylamide or hydro soluble polymers is the initiation of polymerization with Ce^4+^ ions using an alcohol as reducing agent. By selecting monomer bearing alpha alcohol function, biodegradation may be observed (hybrid polymers of biodegradable moieties). Alcohols with weak links (such as azo components) are also prone to degradation after heating. The second promising process is the use of radical ring opening polymerization, along with cyclic ketene acetal monomers. The integration of hydrolysable functional groups in the main chain is efficient to fragment the polymer. This technology is still at the laboratory scale, and more academic research is mandatory to reach an industrial feature. Finally, the copolymerization with monomers inducing biodegradability seems to be an easy way to provide enough degradation to the polymers. However, the choice of monomers is limited, and vinyl acetate seems to be the sole available monomer for this purpose.

## 7. Conclusions

100 years after the revolutionary publication of Staudinger on macromolecules [205], the associated branch of chemistry has accomplished much and enabled the manufacturing and use of life-changing molecules for every inhabitant of the planet, among which are water-soluble polymers used to treat wastewater. The current global energy and climate crisis has triggered the need to review the life cycle of these polymers to see how the global environmental footprint could be reduced, from raw material sourcing to the end of life in the environment. The emerging trends consist of replacing fossil-based raw materials with bio-sourced or recycled ones when and where possible as well as incorporating cleavable monomers into the polymer chains to favor biodegradability and anticipate the regulatory requirements regarding the fate of these molecules in the environment. With a close look at the manufacturing process of polyacrylamides, it is possible to identify the main CO_2_-emitting stages (drying, for instance) and work at improving the reaction and process efficiencies and the recovery of heat for further use, for example.

We also showed that the handprint and benefits of using water-soluble polymers was often overlooked. By allowing either a more efficient use or a reuse of existing resources such as water or by helping develop sustainable agriculture, the polyacrylamides contribute greatly to decreasing the global anthropic footprint. This aspect is non-negligible when calculating the global impact of manufacturing water-soluble polymers.

Finally, given the nature of polymers and their concentration in the different applications mentioned, circular economy or recycling cannot be applied. It is therefore necessary to assess the fate of said polymers in the environment and use macromolecular engineering tools to favor biodegradability to match the existing requirements but also to propose improvements to the existing assessment procedures to provide fair results.

One hundred years after the revolutionary publication of Staudinger on macromolecules, much has been achieved, but much remains to be accomplished. The momentum is right to continue the investigations on how manufacture water-soluble polymers in an even more sustainable way.

## Figures and Tables

**Table 1:** Comparison of CO_2_ emissions during dewatering according 3 cases: without polymer, with fossil-based polymer and with ISCC+ certified polymer.

Emissions (in kg CO_2_e·ton^−1^ of Total Solid)	Heat with Natural Gas	Polymer Production	Polymer Dissolution	Total	Emissions Reduction (%)
Without polymer	140	/	/	140	/
With polymer	/	17.5	0.4	17.9	87
With ISCC+ polymer	/	3.2	0.4	3.6	97

**Table 2:** Overview of OECD biodegradation tests.

Biodegradation Test	OECD Guideline	Pass Level	Incubation Conditions	Chemical Concentration	Inoculum Source	Test Duration
Ready biodegradability tests (screening tests)	OECD 301 A	70% DOC removal	Aerobic	10–40 mg DOC·L^−1^	Activated sludge, sewage effluents, surface waters, soils or mixture of these	28 days
	OECD 301 B	60% ThCO_2_		10–20 mg DOC·L^−1^		
	OECD 301 C	60% ThOD		100 mg·L^−1^	Fresh samples from sewage treatment works, industrial WWTPs, soils, lakes orseas, mixed thoroughly together	
	OECD 301 D			2–10 mg·L^−1^ or 5–10 mg ThOD·L^−1^	Derived from secondary effluent of WWTP or laboratory-scale unit, predominantly domestic sewage, alternatively surface water, e.g., river or lake	
	OECD 301 E	70% DOC removal		10–40 mg DOC·L^−1^	Derived from secondary effluent of WWTP or laboratory-scale unit, predominantly domestic sewage	
	OECD 301 F	60% ThOD		100 mg·L^−1^ or 50–100 mg ThOD·L^−1^	Activated sludge, sewage effluents, surface waters, soils or mixture of these	
Inherent (potential) biodegradability tests	OECD 302 A	>20% ThBOD, ThDOC removal or ThCOD (primary biodegradation; >20% Th BOD, ThDOC removal or ThCOD (ultimate biodegradation)	Aerobic	2 –10 mg·L^−1^	Mixed settled sludges after two week aeration period	Not defined
	OECD 302 B			50–400 mg DOC·L^−1^	Activated sludge	
	OECD 302 C			30 mg·kg^−1^	Activated sludge	
Biodegradability in seawater	OECD 306 Shake flash method	70% DOC removal	Aerobic	50–40 mg DOC·L^−1^	Natural seawater (after filtration)	60 days (can be extended)
	OECD 306 closed bottle method	60% ThOD		2–10 mg test substance·L^−1^		28 days (can be extended)

## Data Availability

No new data were created or analyzed in this study. Data sharing is not applicable to this article.

## Abbreviations

AIBN	Azo-Bis-IsobutyroNitrile
ATRP	Atom Transfer Radical Polymerization
BFR	Bundesinstitut für Risikobewertung
CAGR	Compound Annual Growth Rate
CAN	Ceric Ammonium Nitrate
CCS	Carbon Capture and Storage
CEFAS	Centre for Environment, Fisheries and Aquaculture Science
CKA	Cyclic Ketene Acetal
COVID19	Corona Virus Disease 2019
ECHA	European Chemical Agency
EOR	Enhanced Oil Recovery
ICCA	International Congress and Convention Association
IPN	Interpenetrated Network
ISCC+	International Sustainability and Carbon Certification
KPS	Potassium Persulfate
LAMs	Less Active Monomers
LCA	Life Cycle Assessment
MAMs	More Active Monomers
MBA	Methylene-Bis-Acrylamide
NFS	National Sanitation Foundation
Nhase	Nitrile hydratase
NMP	Nitroxide-Mediated Polymerization
OECD	Organization for Economic Co-operation and Development
PAA	Poly(acrylic acid)
PAM	Poly(acrylamide)
PVA	Poly(vinyl alcohol)
RAFT	Reversible Addition–Fragmentation chain-Transfer polymerization
r-ROP	Radical Ring Opening Polymerization
RSB	Roundtable on Sustainable Biomaterials
RSPO	Roundtable on Sustainable Palm Oil
SAP	Super Absorbent Polymer
TARO	Thiocarbonyl Addition Ring Opening
UNESCO	United Nations Educational, Scientific, and Cultural Organization
VA-086	2,2′-Azobis[2 -methyl-*N*-(2-hydroxyethyl)propionamide]
WBCSD	World Business Council for Sustainable Development
